# Caspase-1 inhibitor ameliorates experimental autoimmune myasthenia gravis by innate dendric cell IL-1-IL-17 pathway

**DOI:** 10.1186/s12974-015-0334-4

**Published:** 2015-06-14

**Authors:** Cong-Cong Wang, Heng Li, Min Zhang, Xiao-Li Li, Long-Tao Yue, Peng Zhang, Yue Zhao, Shan Wang, Ruo-Nan Duan, Yan-Bin Li, Rui-Sheng Duan

**Affiliations:** Department of Neurology, Shandong Provincial Qianfoshan Hospital, Shandong University, Jinan, Shandong 250014 China; Central Laboratory, Shandong Provincial Qianfoshan Hospital, Shandong University, Jinan, Shandong 250014 China; School of Medicine, Shandong University, Jinan, Shandong 250012 China

**Keywords:** Caspase-1 inhibitor, IL-1β, Dendric cell, Th17 cell, Follicular helper T cell, Experimental autoimmune myasthenia gravis

## Abstract

**Background:**

IL-1β has been shown to play a pivotal role in autoimmunity. Cysteinyl aspartate-specific proteinase-1 (caspase-1) inhibitor may be an important drug target for autoimmune diseases. However, the effects of caspase-1 inhibitor on myasthenia gravis (MG) remain undefined.

**Methods:**

To investigate the effects of caspase-1 inhibitor on experimental autoimmune myasthenia gravis (EAMG), an animal model of MG, caspase-1 inhibitor was administered to Lewis rats immunized with region 97–116 of the rat AChR α subunit (R97-116 peptide) in complete Freund’s adjuvant. The immunophenotypical characterization by flow cytometry and the levels of autoantibody by ELISA were carried out to evaluate the neuroprotective effect of caspase-1 inhibitor.

**Results:**

We found that caspase-1 inhibitor improved EAMG clinical symptom, which was associated with decreased IL-17 production by CD4^+^ T cells and γδ T cells, lower affinity of anti-R97-116 peptide IgG. Caspase-1 inhibitor decreased expression of CD80, CD86, and MHC class II on DCs, as well as intracellular IL-1β production from DCs. In addition, caspase-1 inhibitor treatment inhibited R97-116 peptide-specific cell proliferation and decreased follicular helper T cells relating to EAMG development.

**Conclusions:**

Our results suggest that caspase-1 inhibitor ameliorates experimental autoimmune myasthenia gravis by innate DC IL-1-IL-17 pathway and provides new evidence that caspase-1 is an important drug target in the treatment of MG and other autoimmune diseases.

## Background

Myasthenia gravis (MG) is an antibody-mediated, T cell-dependent, complements involved autoimmune disease [1, 2], characterized by loss of acetylcholine receptor (AChR) on the postsynaptic membrane of neuromuscular junction and resulted in impaired neuromuscular transmission and muscle weakness. Experimental autoimmune myasthenia gravis (EAMG) can be induced in Lewis rats by immunization with torpedo acetylcholine receptor (TAChR) or a synthetic peptide corresponding to region 97–116 of the rat AChR α subunit (R97-116 peptide), which is a reliable model for human MG and is suitable for investigating the development of novel therapeutic strategies. CD4^**+**^ T helper cells involving Th1, Th2, Th17, and T regulatory (Treg) cells have been shown to play a crucial role in the pathogenesis of EAMG since they assist autoreactive B cells to produce anti-AChR antibodies.

IL-1β is a highly active pro-inflammatory cytokine produced by limited cells, such as dendritic cells, tissue macrophages, and blood monocytes, depending on the activation of caspase-1 [3]. Both pro-IL-1β and pro-IL-18 as inactive precursors are cleaved by the active caspase-1 into functional forms [4, 5]. Caspase-1 itself is cysteine protease, and pro-caspase-1 is cleaved into active caspase-1 in the inflammasome [6].

IL-1β and IL-18 can induce the secretion of IL-17 from many cells of the innate immune system, including γδ T cells and invariant natural killer T (iNKT) cells [7, 8]. Mature IL-1β binds first to the type 1 IL-1 receptor (IL-1RI) on their cell surface and forms the receptor complex with IL-1R accessory protein (IL-1RAcP) [9, 10]. The Toll-IL-1 receptor (TIR) domains of receptor complex recruits the adaptor molecule MyD88, consequently, NF-κB is phosphorylated. Phosphorylated NF-κB translocates to the nucleus and induces the transcription of pro-inflammatory cytokines [11]. IL-1-targeted drugs, such as the IL-1 receptor antagonist anakinra and the neutralizing monoclonal anti-IL-1β antibody canakinumab, result in a rapid and sustained remission in inflammatory diseases [12]. Daily injections of IL-1 receptor antagonist (IL-1ra) decreased the severity of clinical EAMG in C57BL/6 mice with suppressed serum IFN-γ, TNF-α, IL-1β, C3, and anti-AChR IgG1 [13].

It has recently been reported that caspase-1 is critical for the induction of IL-17 production in innate immune cells. The role of caspase-1 in EAE pathogenesis has been demonstrated, and caspase-1 protein and activity were upregulated during the inflammatory stages of the disease. Caspase-1^−/−^ mice were less susceptible to EAE, and this was associated with impaired Th1 cell development and reduced perivascular infiltrates in the CNS. Inhibition of caspase-1 in vivo suppressed the development of Th17 cells and the induction of EAE, and this was reversed by administration of IL-1β or IL-18, more dramatic with IL-1β [14].

In addition, some studies have confirmed that the activated caspase-1 could lead to an upregulation of MHC class II, CD80, and CD86 on DC through the secretion of IL-1β [15]. We addressed the hypothesis that caspase-1 may be an important drug target for autoimmune diseases.

In this study, we examined the role of caspase-1 inhibitor on innate DC IL-1-IL-17 pathway, provided a potential therapeutic strategy to MG.

## Methods

### Animals and reagents

Female Lewis rats weighing 160–180 g (age from 6 to 8 weeks) were purchased from Vital River Laboratories (Beijing, China) and kept at the local animal house under specific pathogen-free conditions, with standard rat chow and water ad libitum. All experiments were approved by the guidelines of the Animal Ethics Committee of Shandong University. Minimum number of animals was used to result in meaningful interpretation of data, and animal pain and discomfort was kept to a minimum level. The caspase-1 inhibitor Ac-YVAD-cmk was purchased from Cayman Chemical (Ann Arbor, Ml, USA), and Recombinant Rat IL-1β was purchased from Peprotech (Rocky Hill, NJ, USA). R97-116 peptide (DGDFAIVKFTKVLLDYTGHI) was synthesized by CL BIO-SCIENTIFIC CO. LTD. (Xian, China).

### Elicitation of EAMG

Rats were injected subcutaneous into both hind foot pads with 200-μl inoculum containing 50 μg R97-116 peptide, 1 mg. Mycobacterium tuberculosis (strain H37RA; Difco, Detroit, MI, USA) in incomplete Freund’s adjuvant (Sigma-Aldrich, St. Louis, MO, USA) on day 0 and were boosted with the same dose along the back on day 11 after the first immunization.

Rats were randomly divided into respective groups (6 rats/group). From day 13 after the first immunization, rats in the Ac-YVAD-cmk treatment group (at doses of 100 μg/rat in a volume of 0.4-ml PBS) were administered intraperitoneally (i.p.) every second day. Rats in the Ac-YVAD-cmk with IL-1β group were injected i.p. with Ac-YVAD-cmk (at doses of 100 μg/rat in a volume of 0.4 ml PBS) from day 13 after the first immunization and IL-1β (at doses of 0.4 μg/rat in a volume of 0.4-ml PBS) from day 14 after the first immunization every second day. EAMG rats were administered with vehicle (PBS) at the same time points.

### Clinical evaluation

Clinical scoring was based on the presence of tremor, hunched posture, muscle strength, and fatigability in a double-blind fashion. Fatigability was assessed after exercise (repetitive paw grips on the cage grid) for 30 s. Disease severity was graded as follows [16]: 0, normal strength and no abnormalities; 1, mildly decreased activity and weak grip or cry, more evident at the end of exercise; 2, clinical signs present before exercise (tremor, head down, hunched posture, and weak grip); 3, severe clinical signs present before exercise, no grip moribund; 4, dead. Rats with intermediate signs were assigned grades of 0.5, 1.5, 2.5, or 3.5, respectively. Results were expressed as the mean score for each group at each time point.

### DC preparation

Spleen mononuclear cell (MNC) suspensions from Lewis rats were prepared by grinding spleen through a cell strainer (Becton Dickenson, Franklin Lakes, NJ, USA) in medium, then depleted of erythrocytes with osmotic lysis. Cells were incubated in 25 mm^2^ Falcon culture flasks (Becton Dickinson). After 1 h 40 min, non-adherent cells were gently removed. New RPMI 1640 (containing 2.05 mM glutamine, HyClone, Beijing, China) medium containing 1 % (*v*/*v*) penicillin-streptomycin (Shandong Lukang Cisen, Jining, China) and 10 % fetal bovine serum (FBS; Gibco, Grand Land, NY, USA) were added to the flasks. After 18 h of incubation, non-adherent DC were collected. In brief, bone marrow cells prepared from femurs and tibias were cultured in RPMI 1640 medium supplemented with 1 % (*v*/*v*) penicillin-streptomycin (Shandong Lukang Cisen), 10 % (*v*/*v*) fetal bovine serum (Gibco), 10 ng/ml of GM–CSF (Peprotech) and 10 ng/ml IL-4 (Peprotech). After a total culture time of 8 days, non-adherent DC were collected. The DC-enriched population contained 75–85 % DC by staining with OX62 mAb, which specifically expressed on rat DC. DC were cultured with LPS (100 ng/ml, Sigma-Aldrich), with or without the caspase-1 inhibitor Ac-YVAD-cmk (8 μM, Cayman Chemical) for 48 h. Then DC were collected, and CD80, CD86, MHC class II, and IL-1β were determined by flow cytometric analysis.

### Flow cytometric analysis of DC

For phenotypic analysis, DCs were washed with 0.5 % bovine serum albumin (BSA; Sigma-Aldrich) in PBS and stained with PE-conjugated anti-rat CD80 (BioLegend, San Diego, CA, USA), FITC-conjugated anti-rat CD86 (BioLegend), and FITC-conjugated anti-rat MHC class II monoclonal antibodies (eBioscience, San Die go, CA, USA) for 30 min at 4 °C in the dark, respectively.

### Preparation of lymph node mononuclear cells

Mononuclear cell (MNC) suspensions from inguinal lymph nodes were prepared by grinding through cell strainers in serum-free medium. Then, cells were washed three times and resuspended to 2 × 10^6^/ml in RPMI 1640 (HyClone) supplemented with 1 % (*v*/*v*) penicillin-streptomycin (Shandong Lukang Cisen) and 10 % (*v*/*v*) fetal bovine serum (Gibco) for the following experiments.

### Flow cytometric analysis of lymph node MNC

After extracellular staining for surface with PerCP-conjugated anti-rat CD3 (eBioscience), PE-conjugated anti-rat CD4 (BioLegend), PE-conjugated anti-rat γδ TCR (eBioscience), and PE-conjugated anti-rat OX62 (BD Biosciences, San Jose, CA, USA), lymph node mononuclear cells were then fixed with 2 % paraformaldehyde for 20 min at 4 °C and permeabilized with 0.5 % saponin and stained intracellularly with FITC-conjugated anti-rat IL-17 (eBioscience) and FITC-conjugated anti-rat IL-1β (Abcam, San Francisco, CA, USA). Treg cells were identified by extracellular staining with FITC-conjugated anti-rat CD4 (eBioscience), PE-conjugated anti-rat CD25 (eBioscience), after fixation and permeabilization, PE-Cy5-conjugated anti-mouse/rat Foxp3 antibodies (eBioscience) was used for staining according to the protocol recommended. Follicular helper T (Tfh) cells were defined by extracellular staining with PE anti-rat-CD4 (BioLegend), PE-Cy7 anit-rat-ICOS (eBioscience), and FITC anti-rat-CXCR5 (Abcam). Then, samples were analyzed within 24 h with a BD FACScan (BD Biosciences) using Cell Quest software (BD Biosciences).

### Cell proliferation assay

Cell viability was assessed by measuring the conversion of the trazolium salt WST-8 to formazan according to the manufacturer’s instructions [Cell Counting Kit-8 (CCK-8); Dojindo, Kumamoto, Japan] [17]. Briefly, MNC suspended in 200-μl aliquots containing 4 × 10^5^ cells were cultured in triplicates in flat-bottomed 96-well microtitre plates (Corning, NY, USA) in the absence or presence of R97-116 peptide (10 μg/ml). Negative controls were incubated with RPMI 1640 (HyClone). After 72 h of incubation in a humidified atmosphere of 95 % air and 5 % CO_2_ at 37 °C, the cells were incubated with 10 μl CCK-8 for 4 h at 37 °C. Then, the absorbance was read at 450 nm subtracted from 630 nm on a microplate reader and results were expressed as OD values ± SD.

### Cytokine assay by ELISA

Supernatants were removed after 72 h of culture, and IL-1β (eBioscience) or IL-17 (BioLegend) concentrations were determined by sandwich ELISA kits according to the manufacturer’s instructions. Supernatant samples and standard samples were added to the wells and incubated for 2 h at room temperature. After washing, the plates were incubated for 1 h with detection antibody solution. Then, Avidin-HRP solution was added, incubated at room temperature for 30 min. Plates were followed by development with substrate solution. Finally, OD value into each well was measured at 450 nm subtracted from 570 nm using a microplate ELISA reader. Determinations were performed in duplicate and the results were expressed as picograms per milliliter. Results were expressed as mean OD value of samples ± SD.

### Detection of serum anti-R97-116 peptide IgG antibody by ELISA

Microtitre plates (Coaster, Cambridge, MA, USA) were coated with R97-116 peptide (5 μg/ml) at 4 °C over night. Then, the plates were blocked with 200 μl of PBS containing 0.05 % Tween 20 and 10 % FBS at 37 °C for 1.5 h. Serum samples (1:100 in PBS/0.05 % Tween 20) were added to the wells and incubated for 2 h at 37 °C. After washing, the plates were incubated for 1 h with biotinylated rabbit anti-rat IgG (1:3000; Biosynthesis Biotechnology, Beijing, China). Then, streptavidin-horseradish peroxidase (1:1000; Biosynthesis Biotechnology) was added, incubated at 37 °C for 30 min. Plates were washed with PBS containing 0.05 % Tween 20 and followed by development with tetramethylbenzidine (TMB) substrate (Tiangen Biotechnology, Beijing, China). Finally, OD value into each well was measured at 450 nm subtracted from 630 nm using a microplate ELISA reader. Each serum was tested in triplicate. Results were expressed as mean OD value of samples ± SD.

### Detection of relative affinity of serum anti-R97-116 peptide IgG antibody by ELISA

The relative affinity of anti-R97-116 antibodies was determined by ELISA using thiocyanate elution [18]. Briefly, microplates (Coaster) were coated with R97-116 peptide (5 μg/ml) and uncoated sites were blocked with 10 % FBS (Gibco). Diluted serum with a predetermined amount of anti-R97-116 antibodies was added and incubated. Then, 200 μl of appropriate quantities of potassium thiocyanate (KSCN) were added in duplicate and incubated at room temperature for 15 min, followed by biotinylated rabbit anti-rat IgG and streptavidin-horseradish peroxidase. The color was developed with TMB and expressed as OD value at 450 nm subtracted from 630 nm. The relative affinity is expressed as affinity index, equal to the molarity of KSCN resulting in 50 % of the absorbance obtained in the absence of KSCN.

### Statistical analysis

The SPSS 17.0 computer program (SPSS Inc., Chicago, IL, USA) was used for all calculations and statistical evaluations. Differences between two groups were tested by two-tailed Student *t* test and among three groups by one-factor analysis of variance (ANOVA) followed by least significant difference (LSD) test as a post hoc test. Results were presented as means ± SD, and a level of *p* < 0.05 was considered significant.

## Results

### Effects of caspase-1 inhibitor on the phenotype and intracellular cytokines of DCs in vitro

**Fig. 1:**
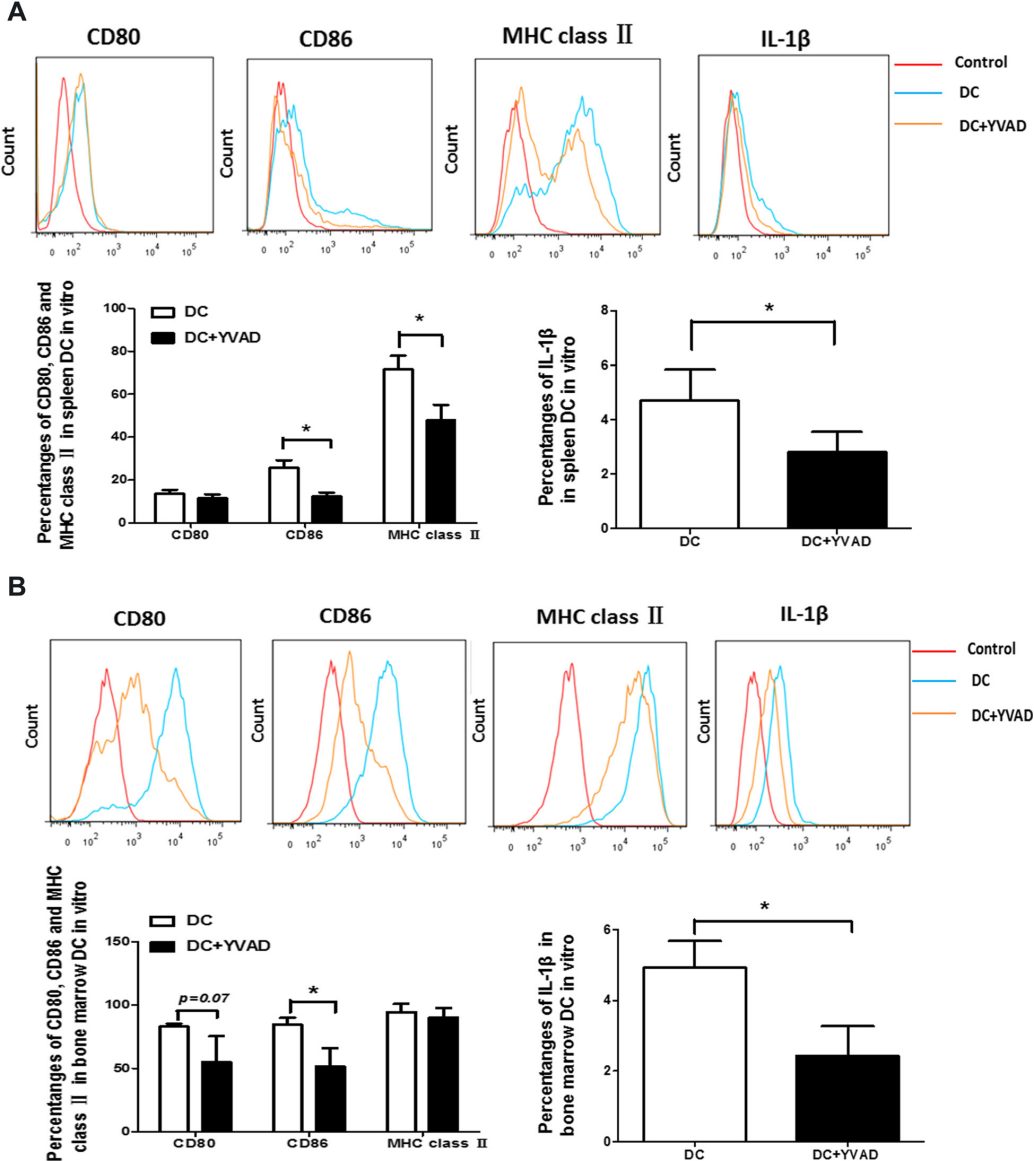
Effects of caspase-1 inhibitor on the phenotype and IL-1β of DCs in vitro. DC from Lewis rats were stimulated with LPS (100 ng/ml) and cultured with the caspase-1 inhibitor Ac-YVAD-cmk (8 μM) for 48 h. Expression of CD80, CD86, MHC class II, and IL-1β from spleen DC (**a**) and bone marrow DC (**b**) were assessed by FACS. Data are expressed as mean ± SD of *n* = 3 rats/group representative of three independent experiments (*p < 0.05)

To explore whether caspase-1 inhibitor could suppress the maturation of DCs, DCs were cultured with or without Ac-YVAD-cmk, and the expression of CD80, CD86, and MHC class II on DCs were analyzed by flow cytometry. The phenotypic analysis of spleen DCs showed that the expression of CD86 and MHC class II were inhibited by caspase-1 inhibitor (*p* < 0.05, respectively) in vitro. Furthermore, the intracellular IL-1β from spleen DCs cultured with or without Ac-YVAD-cmk in vitro was detected by flow cytometry. The results showed that IL-1β production was decreased by caspase-1 inhibitor in vitro (Fig. 1a). The phenotypic analysis of bone marrow DCs showed that the expression of CD80 and CD86 were inhibited by caspase-1 inhibitor, and IL-1β production was also decreased (Fig. 1b).

### Caspase-1 inhibitor suppresses the development of EAMG and regulates the phenotype of DC in EAMG

**Fig. 2:**
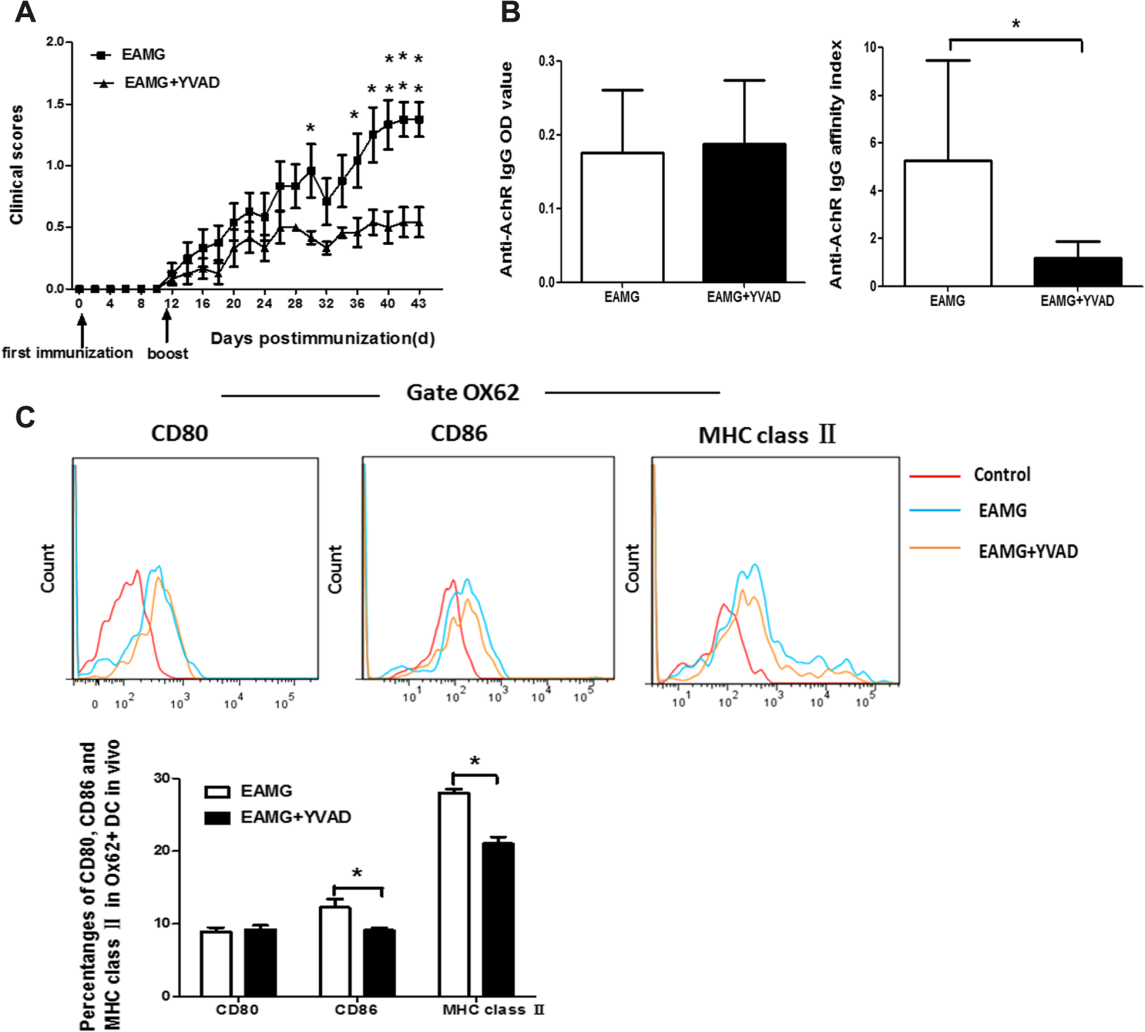
Caspase-1 inhibitor ameliorated EAMG severity and decreased the expression of CD86 and MHC class II among OX62^+^DC in EAMG rats. The rats in Ac-YVAD-cmk treatment group exhibited lower clinical scores when compared with rats in EAMG group (**p < 0.01) (**a**). The serum was obtained on day 43 p.i. and anti-R97-116 IgG titer and affinity were determined (**b**). MNCs were isolated from the lymph nodes of rats in EAMG and Ac-YVAD-cmk groups. Expression of CD86 and MHC class II among OX62^+^DC were decreased in rats treated with Ac-YVAD-cmk compared to that in EAMG rats in vivo (*p < 0.05) (**c**)

To address the role of caspase-1 inhibitor in Lewis rats with ongoing EAMG, the EAMG rats were injected i.p. with caspase-1 inhibitor Ac-YVAD-cmk every second day from day 13 after the first immunization. The rats in Ac-YVAD-cmk treatment group exhibited lower clinical scores when compared with rats in EAMG group. On the day of the experiment termination, the clinical scores of the rats in Ac-YVAD-cmk treatment group averaged 0.54 ± 0.29, while the EAMG group developed more severe symptom, and the clinical scores averaged 1.37 ± 0.34 (*p* < 0.01) (Fig. 2a). Serum was collected on day 43 p.i. to determine anti-R97-116 peptide IgG production by ELISA. There was no difference for the levels of anti-R97-116 IgG between Ac-YVAD-cmk and EAMG groups. However, the affinity in Ac-YVAD-cmk group was lower than that in EAMG group (Fig. 2b). Further, the percentages of CD86 and MHC class II positive cells among OX62^+^DC were significantly (*p* < 0.05) decreased in rats treated with Ac-YVAD-cmk compared to that in EAMG rats in vivo (Fig. 2c).

### Effects of exogenous IL-1β on the caspase-1 inhibitor in EAMG

**Fig. 3:**
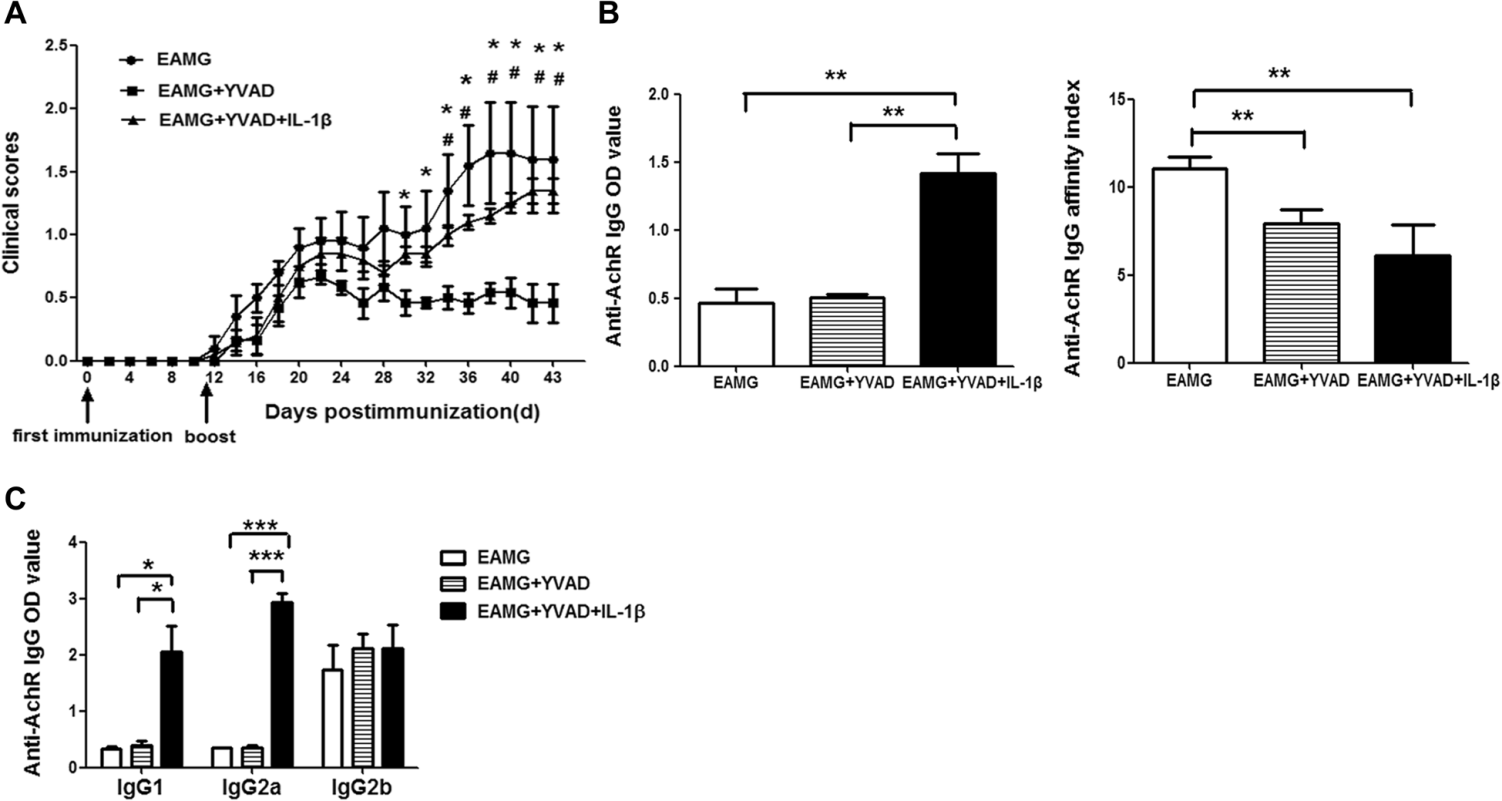
Administration of exogenous IL-1β in vivo aggravated the severity of EAMG rats treated with Ac-YVAD-cmk. The rats treated with Ac-YVAD-cmk showed lower clinical scores from day 30 to 43 p.i. compared with rats in Ac-YVAD-cmk with IL-1β group (^#^ *p* < 0.05) and EAMG group (*p < 0.05) (**a**). Anti-R97-116 peptide IgG titers and the affinity index of anti-R97-116 peptide IgG were determined by ELISA, and results were determined by OD values at 450 nm subtracted from 630 nm (**b**). Anti-R97-116 peptide IgG1, IgG2a, and IgG2b titers were determined by ELISA, and results were determined by OD values at 450 nm subtracted from 630 nm (**c**). Serum was obtained on day 43 p.i. All results are expressed as means ± SD (*n* = 6 in each group) (*p < 0.05, **p < 0.01, and ***p < 0.001)

To investigate the mechanism of caspase-1 inhibitor on EAMG, the EAMG rats were injected i.p. with caspase-1 inhibitor Ac-YVAD-cmk and IL-1β. The rats in Ac-YVAD-cmk with IL-1β group exhibited higher clinical scores when compared with rats in Ac-YVAD-cmk treatment group (*p* < 0.05, from day 34 to day 43 p.i.). Moreover, the clinical symptoms between Ac-YVAD-cmk with IL-1β group and EAMG group did not differ significantly (Fig. 3a). The results of IL-1β on humoral immune responses showed that the level of anti-R97-116 IgG in rats treated with Ac-YVAD-cmk with IL-1β was dramatically increased compared with the other two groups (*p* < 0.01 for both comparisons). Lower affinity indexes of anti-R97-116 peptide IgG in two treated groups was observed compared with that in EAMG rats (*p* < 0.01 for both comparisons). Together, the above results suggested that caspase-1 inhibitor decreased the affinity of anti-R97-116 peptide IgG in EAMG rats and exogenous IL-1β increased the production of anti-R97-116 antibody without improving the affinity (Fig. 3b). Furthermore, there was no difference for the levels of anti-R97-116 IgG1, IgG2a, and IgG2b between EAMG group and Ac-YVAD-cmk group. The level of anti-R97-116 IgG1 and G2a in rats treated with Ac-YVAD-cmk with IL-1β was dramatically increased compared with the other two groups (*p* < 0.05, *p* < 0.001, respectively) (Fig. 3c).

### Caspase-1 inhibitor attenuates EAMG via suppressing DC IL-1β, CD4^+^ T and γδ T cells IL-17 pathways

**Fig. 4:**
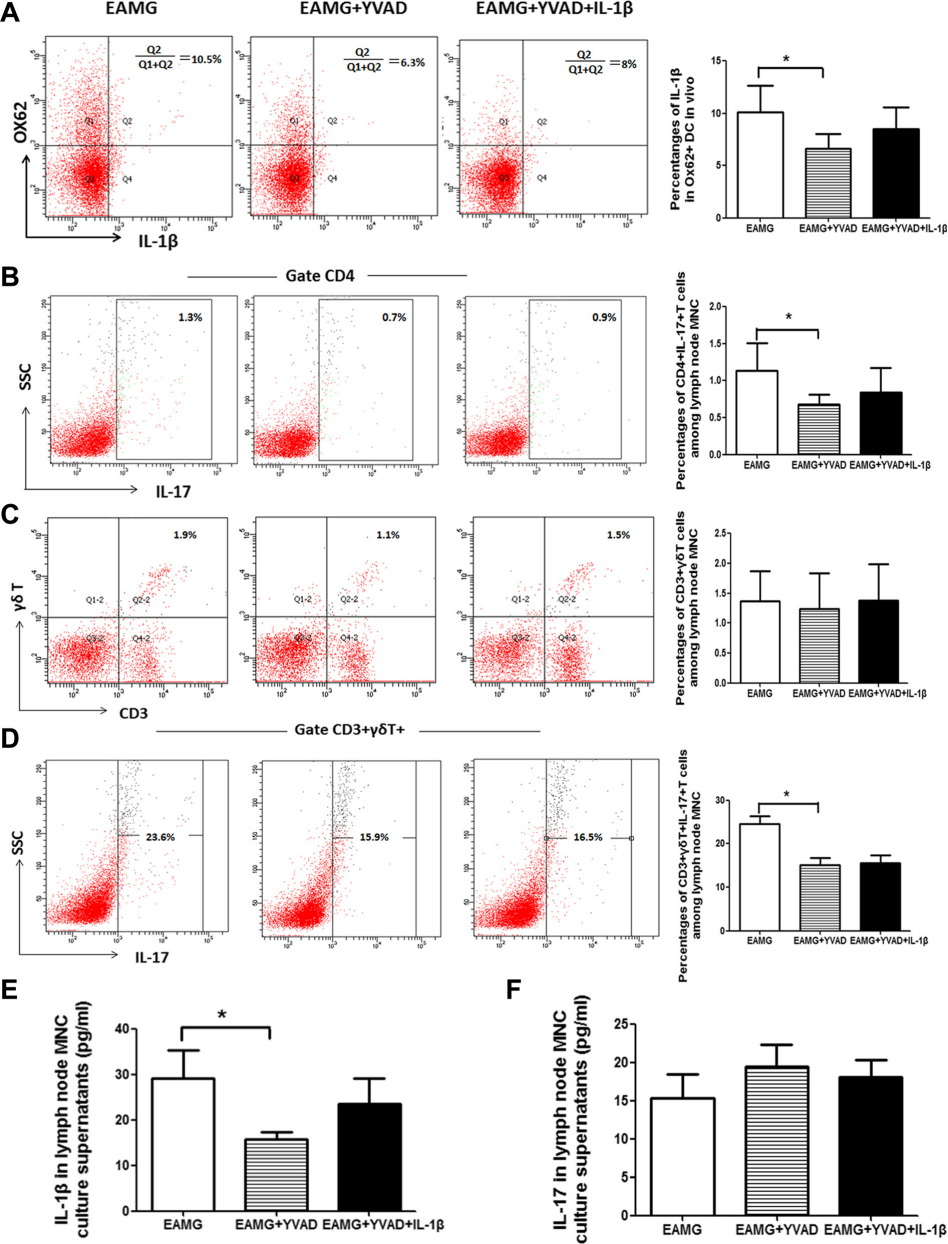
Potential mechanism underlying the effects of caspase-1 inhibitor on EAMG. MNCs were isolated from the lymph nodes of rats in respective groups. Expression of IL-1β positive cells among OX62^+^ DC was assessed by FACS as indicated (**a**). Cells were stained for surface γδ TCR and CD4 as well as intracellular IL-17 and analyzed by flow cytometry. Results are mean percentages of CD4^+^IL-17^+^ cells (**b**), γδ T cells (**c**), and IL-17^+^ γδ T cells (**d**). On day 43 p.i., lymphocytes were purified from the lymph nodes and co-cultured with R97-116 peptide. Supernatants were harvested after 72 h of co-culture. The levels of IL-1β (**e**) and IL-17 (**f**) were determined by ELISA. Data are expressed as means ± SD (*n* = 6 in each group) (*p < 0.05)

To further investigate the mechanism underlying the effects of caspase-1 inhibitor on EAMG, we detected the intracellular IL-1β, IL-17 production from lymph node by flow cytometry. As shown in Fig. 4a, the percentage of IL-1β positive cells among OX62^+^ DC was reduced in rats treated with Ac-YVAD-cmk when compared with that in EAMG rats (*p* < 0.05), which was partly reversed by IL-1β but not statistically significant (*p* = 0.2). There was no difference between EAMG group and Ac-YVAD-cmk with IL-1β group. Secondly, we detected the intracellular IL-17 production by flow cytometry. The percentage of CD4^+^ IL-17^+^ cells was reduced in rats treated with Ac-YVAD-cmk when compared with that in EAMG rats (*p* < 0.05), which was partly reversed by exogenous IL-1β but not statistically significant (*p* = 0.5) (Fig. 4b). γδ T cells have been shown to be an important source of IL-17 in the course of autoimmune diseases and have a pathogenic role during the development of diseases. In our study, there were no differences among the three groups in terms of the numbers of γδ T cells in MNC from lymph nodes (Fig. 4c). However, the percentage of IL-17-expressing γδ T cells was reduced in rats treated with Ac-YVAD-cmk when compared with that in EAMG rats (*p* < 0.05), and there were no statistical difference between two treatment groups (Fig. 4d). We next examined the levels of IL-1β and IL-17 in culture supernatants of lymphocytes stimulated with R97-116 peptide. The production of IL-1β in the culture supernatants of Ac-YVAD-cmk treated group was reduced when compared with that in EAMG group (*p* < 0.05). The production of IL-1β in the culture supernatants in rats treated with Ac-YVAD-cmk and IL-1β was increased when compared with that in Ac-YVAD-cmk rats, but this difference did not reach to a statistically significant value(*p* = 0.24). There was no difference between EAMG group and Ac-YVAD-cmk with IL-1β group (Fig. 4e). There were no differences for the levels of IL-17 among the three groups (Fig. 4f).

### Effects of caspase-1 inhibitor on lymphocyte proliferation and CD4^+^CD25^+^Foxp3^+^ T, CD4^+^CXCR5^+^ICOS^+^ T cell subset differentiation

**Fig. 5:**
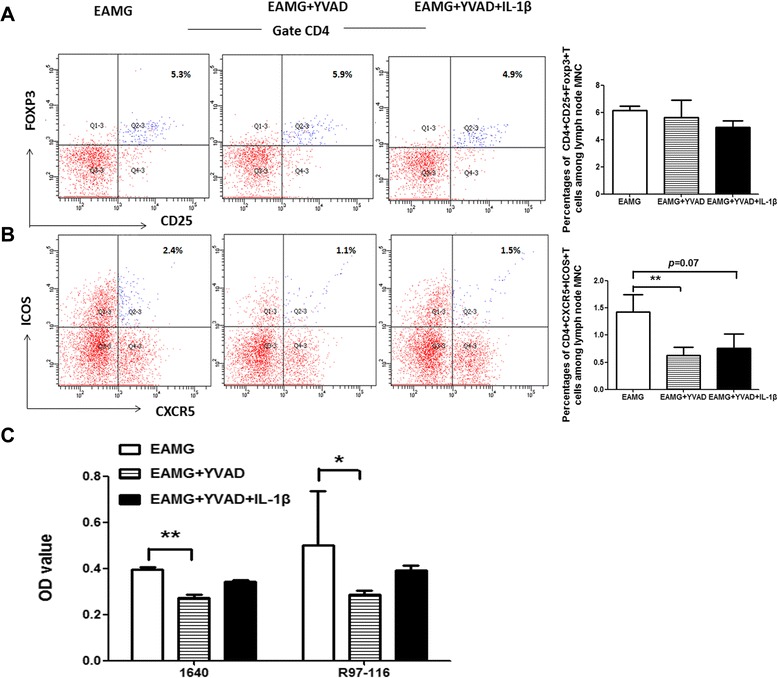
Caspase-1 inhibitor suppressed Tfh cells and lymphocyte proliferation but did not influence Treg cells in EAMG rats. MNCs were isolated from the lymph nodes of rats in three groups. CD4^+^CD25^+^Foxp3^+^ T cells (**a**) and CD4^+^CXCR5^+^ICOS^+^ T cells (**b**) were assessed by FACS as indicated. Proliferation response of lymphocytes derived from three groups were studied on day 43 p.i.. Lymphocyte proliferation was measured by using CCK-8 and OD values at 450 nm subtracted from 630 nm (**c**). The results are expressed as means ± SD (*n* = 6 in each group) (*p < 0.05 and **p < 0.01)

We examined whether the effects of caspase-1 inhibitor was correlated with Treg cells in EAMG rats. The results showed that there was no difference for the percentage of CD4^+^CD25^+^Foxp3^+^ T cells among three groups, respectively, suggesting that caspase-1 inhibitor induced immune suppression rather than by CD25^+^Foxp3^+^ T regular cells in the present study (Fig. 5a). Follicular helper T cells are able to provide essential help to the antigen-specific B cells within the secondary lymphoid organ. The percentage of CD4^+^CXCR5^+^ICOS^+^ Tfh cells in rats treated with Ac-YVAD-cmk decreased compared with that in EAMG rats (*p* < 0.01). The percentage of CD4^+^CXCR5^+^ICOS^+^ Tfh cells in rats treated with Ac-YVAD-cmk with IL-1β exhibited a decreased trend but did not reach the statistically significant value (*p* = 0.07) compared with that in EAMG group. These data suggested that caspase-1 inhibitor may alter humoral immune responses in EAMG associated with influencing Tfh population (Fig. 5b). Lymphocyte proliferation was measured after 72 h of culture in the absence or presence of R97-116 peptide by using the CCK-8 assay. In the absence or presence of R97-116 antigen, lymphocyte proliferation in Ac-YVAD-cmk group was decreased compared with that in EAMG group (*p* < 0.01 and *p* < 0.05, respectively). Meanwhile, we did not find statistical difference between two treatment groups (Fig. 5c).

## Discussion

The new finding of this study is that caspase-1 inhibitor treatment ameliorated EAMG severity. We found that caspase-1 inhibitor suppressed the expression of co-stimulatory molecules CD80, CD86, and MHC class II on the surface of the DCs and the expression of intracellular IL-1β of the DCs. Inhibition of caspase-1 suppressed IL-17 expression by γδ T cells and CD4^+^ T cells. Furthermore, we demonstrated that caspase-1 inhibitor suppressed lymphocyte proliferation, decreased the number of Tfh cell, and the affinity of anti-R97-116 IgG in EAMG rats. The present study provides convincing evidence that caspase-1 inhibitor ameliorates EAMG by innate DC IL-1-IL-17 pathway.

Dendritic cells (DC) are professional antigen-presenting cells (APC), playing a major role in inducing adaptive immune responses and maintaining immunological tolerance. DC is the critical link between innate and adaptive immunity, by means of cellular interactions and secretion of pro-inflammatory and immunoregulatory cytokines [19]. The functional activities of DC depend on their state of activation and maturation. Mature DCs (mDC) can efficiently induce the development of effector T cells to immunity, whereas immature DCs (imDC) can lead to immune tolerance or delay the immune response by inducing T cell anergy and decreasing the T cell proliferation. Typically, mature DCs are associated with increased expression of co-stimulatory molecules CD86 and MHC class II [20, 21]. Stimulation of DCs induced high concentrations of IL-1β [15], and the release of IL-1β could be in the form of autocrine role in DCs itself to promote the maturity of the DCs [22]. Furthermore, DC-based vaccines matured with a gold standard maturation cocktail consisting of IL-1β [23]. TiO_2_ and SiO_2_ nanoparticles could activate caspase-1 and led to an upregulation of MHC class II, CD80, and CD86 on DC, via the secretion of IL-1β [24]. Consistent with these findings, we found that inhibition of caspase-1 decreased the expression of CD80, CD86, and MHC class II of DC, suppressed IL-1β production by DC, and decreased the production of IL-1β in culture MNC supernatants.

In the present study, caspase-1 inhibitor suppressed lymphocyte proliferation in EAMG. Decreased proliferation of R97-116 specific T lymphocytes may contribute to the clinical improvement and immune tolerance induction in EAMG.

Th17 cells, CD4^+^ T cells that secrete IL-17, are pathogenic in autoimmune diseases, and their differentiation and expansion are driven by the cytokines IL-6 and TGF-β [25–27]. Furthermore, it has recently been reported that IL-1 signaling is critical for Th17 cell differentiation [28]. γδ T cells, an unconventional T cell subset which bridges the innate and adaptive immune responses, have been shown to play a pathogenic role early in inflammatory response. γδ T cells can secrete IL-17 during infection, which is independent of IL-6, an essential cytokine for driving the development of Th17 cells [29]. However, recent studies have shown that early IL-17 from γδ T cells may promote the induction or activation of Th17 cells, which requires for IL-1β. The mechanism that innate IL-17 from γδ T cells promotes the induction or activation of Th17 cells might be due to both directly by interacting with IL-17R on CD4^+^ T cell and indirectly by enhancing the production of cytokines and chemokines from DCs to promote the development of Th17 cells. First, IL-17 and IL-21 produced by γδ T cells can promote IL-17 production by Th17 cells [30]. Second, the production of IL-23, IL-1β, IL-6, TGF-β, CCL2, and CXCL10 from DCs promote the expansion and recruitment of Th17 cells. This is consistent with our conclusion that inhibition of caspase-1 suppressed intracellular IL-17 production by γδ T cells and CD4^+^ T cells, although it did not decrease IL-17 level in culture MNC supernatants. Flow cytometry analysis shows the instant intracellular production of IL-17 by CD4^+^ T cells and γδ T cells, but ELISA detects the accumulated levels of IL-17 released by MNCs to the supernatants, which is affected by various factors, like culture time and the volume of culture medium. In addition, a range of immune cell types have all been shown to secrete IL-17 in vivo, and the experimental result in vitro could not fully reflect complex environment in vivo. So, for ELISA analysis, the differences between groups may be diminished by these factors. However, we found that administration of IL-1β in vivo could not reverse the suppressive effects of caspase-1 inhibitor on Th17 cells. Consistent with our findings, previous studies have shown that IL-1β as a single stimulus could not induce IL-17 production, which may require assistance of other cytokines like IL-23 for further study [31, 32]. The more severe disease in Ac-YVAD-cmk with IL-1β group may due to the directly pathogenic role of IL-1β itself compared with the Ac-YVAD-cmk group, which may be independent of IL-17 pathway. In our study, the level of anti-R97-116 IgG in Ac-YVAD-cmk with IL-1β group was dramatically increased compared with that in Ac-YVAD-cmk group, and there were no differences for the affinity indexes of anti-R97-116 peptide IgG in two treated groups. Hence, IL-1β was able to override the effect of caspase-1 inhibitor on clinical severity. Compared with wild-type mice, IL-1β^−/−^ mice suppressed the development of clinical EAMG, by means of reducing levels of serum anti-AChR antibodies and Th1 (IFN-γ, IL-2) and Th2 (IL-4) cytokine responses [33].

The role of IL-1β in antibody production has been reported. IL-1 could increase serum IgE and IgG1 levels when LPS is used as an adjuvant [34]. In an earlier study, carp IL-1β enhanced the humoral antibody response, increasing a major fish pathogen antibody [35]. In the present study, we found that the caspase-1 inhibitor has no effect on anti-R97-116 peptide IgG production, and the level of IgG, IgG1, and IgG2a in the group treated with IL-1β was increased, which was associated with the role for IL-1β in antibody production.

Antibody affinity maturation occurs within the specialized microenvironment of germinal centers (GC). Tfh cells, a recently defined CD4^+^ T cell subset characterized by the expression of the chemokine receptor CXCR5 and the transcriptional factor Bcl-6, provide a help for B cells to enhance antibody responses and undergo antibody affinity maturation in the germinal center [36–39]. Tfh cell differentiation could be divided into two stages: priming and maintenance stages. At the early differentiation of Tfh, DC priming is sufficient to induce Bcl-6 expression and initial Tfh cell differentiation [40]. The inducible regulatory T cells and the tolerogenic DC could inhibit Tfh cells [41]. In our study, caspase-1 inhibitor decreased the number of Tfh cells by inhibiting DC maturation, which may further decrease the affinity of anti-R97-116 peptide IgG.

## Conclusion

Our data suggested that caspase-1 inhibitor can inhibit DC maturation and IL-1β production from DC, resulting in the lower number of Tfh and decreased IL-17 production by γδ T cells and CD4^+^ T cells, decreasing the affinity of anti-R97-116 peptide IgG, thereby suppressing EAMG progression. Administration of exogenous IL-1β in vivo could not reverse the suppressive effects of caspase-1 inhibitor. These data suggested that caspase-1 inhibitor has greater potential for the treatment of EAMG and even human MG.

## Abbreviations

APC: antigen-presenting cell
Caspase: cysteinyl aspartate-specific proteinase
DC: dendric cell
EAMG: experimental autoimmune myasthenia gravis
ELISA: enzyme-linked immunosorbent assay
IL-1RAcP: IL-1R accessory protein
iNKT: invariant natural killer T
KSCN: potassium thiocyanate
MNC: mononuclear cell
TAChR: torpedo acetylcholine receptor
